# A framework to analyze cerebral mean diffusivity using surface guided diffusion mapping in diffusion tensor imaging

**DOI:** 10.3389/fnins.2015.00236

**Published:** 2015-07-14

**Authors:** Oh-Hun Kwon, Hyunjin Park, Sang-Won Seo, Duk L. Na, Jong-Min Lee

**Affiliations:** ^1^Department of Biomedical Engineering, Hanyang UniversitySeoul, South Korea; ^2^School of Electronic and Electrical Engineering, Sungkyunkwan UniversitySuwon, South Korea; ^3^Department of Neurology, Samsung Medical Center, Sungkyunkwan University School of MedicineSeoul, South Korea

**Keywords:** magnetic resonance imaging, diffusion tensor imaging, mean diffusivity, cortical thickness, geometric distortions

## Abstract

The mean diffusivity (MD) value has been used to describe microstructural properties in Diffusion Tensor Imaging (DTI) in cortical gray matter (GM). Recently, researchers have applied a cortical surface generated from the T1-weighted volume. When the DTI data are analyzed using the cortical surface, it is important to assign an accurate MD value from the volume space to the vertex of the cortical surface, considering the anatomical correspondence between the DTI and the T1-weighted image. Previous studies usually sampled the MD value using the nearest-neighbor (NN) method or Linear method, even though there are geometric distortions in diffusion-weighted volumes. Here we introduce a Surface Guided Diffusion Mapping (SGDM) method to compensate for such geometric distortions. We compared our SGDM method with results using NN and Linear methods by investigating differences in the sampled MD value. We also projected the tissue classification results of non-diffusion-weighted volumes to the cortical midsurface. The CSF probability values provided by the SGDM method were lower than those produced by the NN and Linear methods. The MD values provided by the NN and Linear methods were significantly greater than those of the SGDM method in regions suffering from geometric distortion. These results indicate that the NN and Linear methods assigned the MD value in the CSF region to the cortical midsurface (GM region). Our results suggest that the SGDM method is an effective way to correct such mapping errors.

## Introduction

Diffusion tensor imaging (DTI) is a quantitative magnetic resonance imaging (MRI) technique that measures the random motion of water within the tissue microstructure in the brain (Moseley et al., 1991). DTI is considered an important technique for brain microstructural analysis (Basser, 1995; Beaulieu, 2002). Fractional anisotropy (FA) and mean diffusivity (MD) are the most widely used as scalar measurements to characterize diffusion properties. The FA value, a scalar parameter of the degree of anisotropy, is dependent on the directionally constrained anisotropic water diffusion in white matter (WM) related to the packing density of the fiber bundles (Liu et al., 2006). The MD value, a quantitative measure of the mean motion of water, is used mainly to detect pathological changes in the WM (Fellgiebel et al., 2004). The MD value can also describe the microstructural diffusion properties in gray matter (GM) because water diffusivity in this tissue is nearly isotropic (Kubicki et al., 2002; Fellgiebel et al., 2004; Sundgren et al., 2004; Càmara et al., 2007). Several studies have used the MD value in the cortical GM to detect the characteristics of specific diseases. For example, the elevated MD values in the cortical GM were observed in the patients with multiple sclerosis (Vrenken et al., 2006; Zhou et al., 2010), mild cognitive impairment and Alzheimer's disease (Naggara et al., 2006; Ray et al., 2006; Rose et al., 2008; Serra et al., 2010), traumatic brain injury (Turken et al., 2009), and Creutzfeldt–Jakob disease (Liu et al., 2006). Several study have used surface-based DTI analyses to investigate the microstructural properties of cortical GM (Koo et al., 2009; Turken et al., 2009). Cortical surface-based analysis, which generates two estimated surfaces such as GM/WM boundary and GM/cerebrospinal fluid (CSF) boundary, has been widely used in the T1-weighted volume MRI to measure the cortical thickness.

For the cortical surface-based DTI analysis, the MD volumes are first projected to the positions corresponding to the vertex of the mid surface, which is located in the GM regions. Vertex-wise statistics are then calculated to find abnormal regions. Since diffusion-weighted imaging generally suffers from substantial geometric distortions that make it difficult to align the b0 volume onto the T1-weighted volume, poor anatomical correspondence between the non-diffusion-weighted volume (b0 volume) from conventional Echo–Planar Imaging (EPI) and an undistorted, high-resolution anatomical volume (T1-weighted volume MRI) is one of the most critical issues in the cortical surface-based DTI analysis (Hutton et al., 2002). One typical approach dealing with this issue is to utilize linear or non-linear coregistration methods (Jezzard and Balaban, 1995; Smith et al., 2004; Saad et al., 2009). However, Linear method is inaccurate when the EPI image has a large amount of non-linear distortions. The non-linear method might be advantageous in largely non-linearly distorted EPI images (Jenkinson et al., 2002), but still has some drawbacks such as poor robustness (especially when dealing with brain pathologies), difficulty in validation, and long computation times (Brett et al., 2001; Crum et al., 2003; Hutton and Braun, 2003; Gartus et al., 2007). Therefore, it is difficult to address the issue with coregistration methods only.

The next critical issue in the cortical surface-based DTI analysis is the mapping of MD value, which finds a way to transfer the voxel of MD volume to the corresponding vertex of the mid surface. Most previous studies employed the Nearest-Neighbor (NN) method which samples the voxel that is closest to this point or Linear method which samples the MD value with a linearly weighted average of that neighborhood (Turken et al., 2009). Both methods work well only if the anatomical correspondence between the MD volume and T1-weighted volume is guaranteed. For example, the MD value in cerebrospinal fluid (CSF) or WM could be projected to the vertex on the mid surface, which results in an over- or underestimation of the true MD value. Some studies did not consider the geometric distortion, where the coregistration was only achieved by affine or rigid-body registration and the MD volume were projected to the vertex of the mid surface using a NN or Linear method (Turken et al., 2009). However, such registration methods are insufficient to achieve anatomical correspondence between two volumes, which could result in faulty assignment of the MD value (Jezzard and Balaban, 1995; Saad et al., 2009; Villain et al., 2010). Such misalignment would lead to mismatches between tissue boundaries of the cortical surface and the MD volume. For example, the GM/CSF or GM/WM tissue boundary of the MD volume could be inconsistent with the tissue boundary on surface spaces, such as the pial surface or WM surface reconstructed from the T1-weighted MRI scans. If the tissue boundary on the MD volume corresponding to the surface space could be identified correctly, the faulty assignment of the MD value caused by geometric distortion could be minimized. Other studies addressed the misalignment issue by the non-EPI T2^*^ volume, where the geometric distortion causing misalignment was corrected by a field map calculated by two non-EPI T2^*^ volumes (Hutton et al., 2002; Cusack et al., 2003). However, this approach requires the additional acquisition of two non-EPI T2^*^ volumes with different echo times, which cannot be applied retrospectively if a field map has not been acquired (Villain et al., 2010).

**Figure 1:**
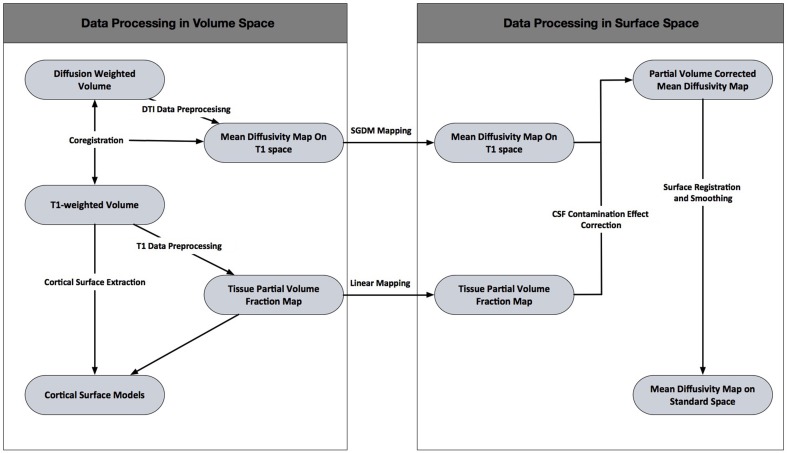
**The outline of the framework for cerebral mean diffusivity analysis**.

In the present study, we propose a novel approach called Surface Guided Diffusion Mapping (SGDM), which compensates for the mapping error caused by misalignment (Figure 1). The purpose of SGDM approach was to compensate for the mapping error. The mapping error originates in misalignment between cortical surface and the MD volume. The cortical surface was reconstructed from T1-weighted volume. These mean that the mapping error arise from misalignment between T1-weighted volume and the MD volume. The mapping error could be rectified in a volume space or surface space. The correction in volume space attempts to improve intracranial correspondence between two volumes using the field map acquisition or fine registration algorithm. If the intracranial correspondence between two volumes is guaranteed, the NN or Linear mapping method would be sufficient. The SGDM approach was not necessary. However, the former requires the additional acquisition of two non-EPI T2^*^ volumes, which cannot be applied retrospectively if a field map has not been acquired. The fine registration algorithm such as non-linear method might be advantageous in largely non-linearly distorted DTI data, but still existed residual misalignments between cortical surface and MD volume. In present study, we tried to correct the mapping error when the MD volume was projected to the cortical surface. We suppose the residual misalignment still existed, though a fine registration algorithm was used.

The SGDM method identified a new GM/CSF boundary in the MD volume corresponding to the pial surface using the MD profile plotted from the voxels overlaid with the vertex of the pial surface toward the line followed by the cortical column. In order to evaluate the performance of the SGDM, coregistration between the b0 volume and T1-weighted volume was performed using the Advanced Normalization Tools-symmetric normalization (ANTs-SyN) (Avants et al., 2008) and affine registration before MD value was projected. The ANTs-SyN is diffeormorphic registration algorithms, which is used to overcome the mis-alignment between T1-weighted and B0 volume. The affine registration is widely used to align two volumes with cost function of mutual information. And then, we compared our SGDM method with results using NN and Linear methods in each registration algorithm.

## Materials and methods

### Data processing in volume space

#### DTI data preprocessing

The DTI data were preprocessed using the Functional MRI of the Brain (FMRIB) Software Library program (Smith et al., 2004). Motion artifacts and eddy current distortions were corrected by normalizing each directional volume to the b0 volume using FMRIB's Linear Image Registration Tool (FLIRT) with 6° of freedom (DOF) (Smith et al., 2004). The diffusion tensor was then calculated using a simple least squares fit of the tensor model to the diffusion, and an MD map was generated.

#### T1 data prerocessing

The native T1-weighted images were corrected for non-uniform intensity resulting from inhomogeneities in the magnetic field (Sled et al., 1998) and normalized into a standardized stereotaxic space using affine transformation (Collins et al., 1994). The corrected and normalized volumes were classified as WM, GM, CSF, or background using an advanced neural network classifier (Zijdenbos et al., 2002). The partial volume effect classification was performed using the trimmed minimum covariance determinant method (Tohka et al., 2004). The partial volume fractions of each tissue class fell between 0 and 1 per voxel.

#### Cortical surface extraction

The cortical surfaces were extracted automatically using the Constrained Laplacian-based Automated Segmentation with Proximities (CLASP) algorithm (MacDonald et al., 2000; Kim et al., 2005). CLASP reconstructs the inner cortical surface by deforming a spherical mesh onto the GM/WM boundary. The outer cortical surface is then expanded from the inner surface to the boundary between the GM and CSF along a Laplacian map. The reconstructed hemispheric cortical surfaces consisted of 81,920 discrete triangular elements (40,962 vertices) forming high-resolution meshes. Because the inner and outer surfaces had the same vertex number and the correspondence of each vertex between surfaces was defined, we reconstructed the cortical midsurface easily and measured the cortical thickness using the t-link method (Lerch and Evans, 2005) of calculating Euclidean distances between linked vertices on both the WM and pial surfaces.

#### Coregistration

To accurately estimate the intracranial correspondence between structural and diffusion weighted spaces, T1-weighted and b0 image were initially skull-stripped using Brain Extraction Tool (Smith, 2002). In present study, we performed coregistration using two algorithms: ANTs-SyN, affine registration. The affine registration is widely used to align two volumes with cost function of mutual information. The ANTs-SyN is one of the diffeomorphic image registration algorithms. It gives relatively better registration performance than affine registration in a variety of T1-weighted MR registration (Klein et al., 2009). Based on the estimated transformation parameter, MD volume was transformed to T1 space for estimating CSF contamination effect and diffusion data mapping.

### Volume to surface space

#### Surface guided diffusion mapping

**Figure 2:**
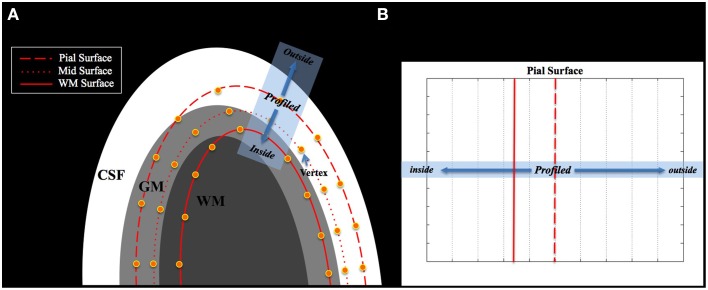
**(A)** Misalignment between the MD image and the cortical surface. The tissue boundary of the MD image does not match the boundary of the cortical surface. **(B)** This shows the profile space obtained from the blue rectangle in **(A)**. The dotted red line is the pial surface and the straight red line is the surface of the WM. The center of the profile is located at the vertex of the pial surface.

NN and Linear methods overlaid the MD volume with a cortical surface line, and projected the MD volume to the vertex closest to the voxel of the MD volume. If the result of coregistration is not accurate, the GM/CSF boundary in the MD volume, where the intensity changes from high (CSF region) to low (GM region), does not correspond with the GM/CSF boundary in surface space (pial surface) as shown in Figure 2A. Under these circumstances, the voxel of the MD volume in the CSF or WM regions might be placed on the vertex of the mid surface, and thus the voxel in CSF or WM region could be projected on the vertex of the mid surface.

In this study, we found the new GM/CSF boundary in the volume space corresponded with the pial surface by plotting the profile of MD values, and estimated the location of the voxel that should be projected to the midsurface vertex using cortical thickness. The details of the SGDM procedure are described below. Note that we used the GM/CSF boundary, not the GM/WM boundary, because of the former's greater contrast in the MD volume.

##### Step 1. definition of the profile direction

We defined the profile direction from the position of the voxel corresponding to the pial surface toward the line followed by the cortical column. This was used to establish the intensity profile of the MD value (see Figure 2A). The center of the profile was located at the vertex of the pial surface. The MD volume was profiled from the center toward the vertex of the WM surface (inside) and toward the opposite direction of the WM surface (outside) as far as the predefined range (see Figure 2B).

##### Step 2. sampling the intensity of the profiles and smoothing the profiles

**Figure 3:**
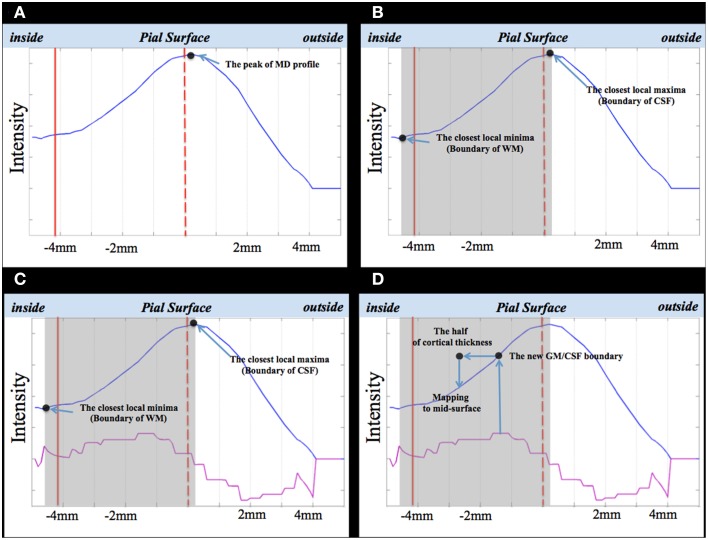
**The SGDM procedure**. Dotted red line is pial surface; straight red line is WM surface; blue line is the smoothed MD profile; pink line, the first differential of the smoothed MD profile **(A–D)**. The gray rectangles in **(B–D)** indicate the search window.

We sampled the intensity of the MD volume along the direction defined in Step 1. Because the profile has a stepwise property, we smoothed the MD using a moving average kernel (see Figure 3A).

##### Step 3. calculating the first differential of MD profiles

As shown in Figure 3, the peak of the MD profile (the black dot in Figure 3A) was placed on the location of the vertex of the pial surface. In this study, we assumed the location of the largest gradient value of the MD profile to be the new GM/CSF boundary, corresponding to the pial surface. We estimated the location of the largest gradient value of the MD profile using the maximum value of the first differential of the MD profile (the purple line in Figure 3C). We used the GM/CSF boundary to estimate the location of the MD profile to be projected to the vertex. For example, if the result of coregistration is accurate, the location of the largest gradient value of the MD profile will be placed on the center of the profile because this point is identical to the point on the pial surface. If the result of coregistration is not accurate, the location of the largest gradient value in the MD profile will be shifted to the left or right from the profile's center. Therefore, when the voxel of the GM region of the MD volume is placed on the CSF region, the location of the largest gradient value of the MD profile is shifted to the right. Conversely, when this voxel is placed on the WM region, the location is shifted to the left. If we assume the location of the largest gradient value of the MD profile to be the new GM/CSF boundary without any constraint, the new GM/CSF boundary can be placed on the adjacent GM or WM region. We needed to restrict the range for searching for the maximum value of the first differential of the MD profile to a specific window to avoid such errors.

##### Step 4. definition of the search window

Since the search window was extended from the WM area to the CSF area, the WM and CSF areas should exist in the same section. The local maxima of the MD profile closest to the vertex of the pial surface formed the boundary of the CSF area (see Figure 3B). We used the local minima of the MD profile to determine the WM area. The local minima of MD profile closest to the boundary of the CSF area corresponded with the central area of the WM. The local minima closest to the peak of MD profile became the boundary of the WM area (see Figure 3B). The window is shown as a gray rectangle in Figure 3B.

##### Step 5. estimation of the new GM/CSF boundary in the MD profile

We calculated the largest gradient value of the MD profile (the maximum of the first differential) in Step 3. The location of the largest gradient value was regarded as the new GM/CSF boundary (see Figure 3D).

##### Step 6. mapping MD values to the vertex of the midsurface

If we can determine the location of the GM/CSF boundary corresponding to the pial surface in the MD profile, we can estimate the location of the MD profile to be projected to the vertex of the mid surface. This was placed on the inside, halfway through the cortical thickness along the MD profile. In Step 5, we estimated the location of the GM/CSF boundary in the MD profile corresponding to the vertex of the pial surface. Therefore, we could determine the location to be projected to the vertex of the mid surface. We projected the voxel of the MD volume to be located halfway into the cortical thickness from the vertex of the mid surface (see Figure 3D).

#### Linear mapping

SGDM method is proposed to compensate for the mapping error caused by misalignment between T1-weighted and MD image. Because the partial volume fraction map was estimated from T1-weighted image, however, the mapping error does not exist while the partial volume fraction map is projected to vertex of cortical surface. Therefore, the SGDM approach was not necessary for mapping the partial volume fraction map to the surface. If partial volume fraction map is obtained from B0 image, the partial volume fraction map should be projected using SGDM method.

### Data processing in surface space

#### Correction of the CSF contamination effect

Since the highly folded, thin structure of the cortical GM (Zilles et al., 1988)in combination with the low resolution of conventional DTI produces significant partial volume effects (Koo et al., 2009), it is important to consider the effect of CSF contamination when analyzing the MD value in the cortical GM. It is critical for distinguishing actual changes in the diffusivity of the GM itself from the changes caused by other effects such as gross morphological changes (Koo et al., 2009).

We projected the voxel of the MD volume to the vertex of the cortical surface using SGDM. Because CSF contaminated the MD value on the vertex, we sought to remove it and to estimate the true MD value in the cortical GM. We estimated the CSF contamination effect as described (Koo et al., 2009). Because the T1-weighted volume has better tissue contrast than the b0 volume, we used the partial volume fraction map generated from the T1-weighted volume to model the CSF contamination effect, rather than using the b0 volume (Koo et al., 2009). Because the MD measured in each vertex depends on the composition of the tissue compartments, it can be represented as the weighted sum of the diffusivity from all tissue compartments, assuming no spin exchange between tissue compartments (Koo et al., 2009). As the MD value of the peripheral WM is known to have a similar range to that of the GM, a two-tissue compartment model was applied here as described (Latour and Warach, 2002; Koo et al., 2009). The CSF contamination in the GM in each vertex on the mid surface was estimated based on Equation (1):

(1) $$\begin{array}{l} \begin{array}{cc} exp(-bD(k)) & =\lambda_{app-gm}(k)\;\cdot \;exp(-bD_{gm}(k)) \end{array}\\ \;+\lambda_{app-csf}(k)\;\cdot \;exp(-bD_{csf}(k)), \end{array}$$

where D is the observed MD value with the CSF contamination effect that was projected to a vertex (k) on the mid surface by SGDM, and D_gm_ and D_csf_ denote the estimated MD values of GM and CSF, respectively. In this study, D_csf_ was set to 3.0 × 10^−3^ mm^2^/s, and *b* is a diffusion-weighting b-value. The expressions λ_app−gm_ and λ_app−csf_ are the apparent signal fraction weightings of the GM and CSF, respectively. They can be expressed as follows:

(2) $$\lambda_{app-i}=\;\frac{\lambda_{i}\;\cdot \;S_{i}(0)}{\sum_{j}\lambda_{j}\;\cdot \;S_{j}(0)},$$

where λ_i_ is the estimated partial volume fraction for each tissue class (λ_GM_ for GM; λ_csf_ for CSF) in a single vertex, which ranges from 0 to 1. S_i_(0), regarded as solely weighted by relaxation, is the signal intensity in the absence of diffusion gradients in a spin–echo sequence. The partial volume fraction map (λ_i_) was projected to the vertex of the mid surface using the NN method. Index j is the total number of tissue classes. S_i_(0) is defined as follows:

(3) $$S_{i}(0)=\rho_{i}\;\cdot \;exp\;\left[\frac{-TE}{T_{2i}}\right]\;\cdot \;\left(1-exp\;\left[\frac{-TR}{T_{1i}}\right]\right),$$

where S_i_(0) is a function of each compartment's equilibrium proton spin density (ρ_i_), T1 is the relaxation time (T_1i_), T2 is the relaxation time (T_2i_), based on the repetition time (TR), and echo time (TE). Finally, λ_app−gm_ was estimated from Equations (2) and (3) for each vertex. We could then estimate the MD value (D_gm_) in the cortical GM without the effect of CSF contamination.

#### Surface registration and smoothing

To compare the MD value of corresponding regions of the surface model between the groups, the MD value was spatially normalized using surface-based 2-D registration. Two-dimensional surface registration used the sphere to sphere warping algorithm. Vertices of each subject are non-linearly registered to an average template (Robbins, 2003). This algorithm was tuned for chosen parameter values, improving the resulting registrations. Using the transformation, MD value on the vertices was transformed to a template. Gaussian kernel smoothing with 20 mm FWHM (full-width half-maximum) was used to increase the signal-to-noise ratio (Chung et al., 2003).

### Evaluation

#### Data set

The data set used to evaluate the SGDM method was collected at the Samsung Medical Center, Seoul, South Korea. Informed, written consent for participation was obtained from each individual, and the Institutional Review Board of the Samsung Medical Center approved the protocol of this study. The participants in this study were 38 healthy adults (18 men and 20 women; mean age ± standard deviation 28.98 ± 3.55 years) who underwent T1-weighted volume MRI and diffusion-weighted MRI. Three-dimensional T1-weighted, spoiled gradient-echo MRI scans were acquired using a 3T MRI scanner (GE Signa, Milwaukee, WI, USA) with the following imaging parameters: coronal slice thickness, 1.5 mm; echo time (TE), 7 ms; repetition time (TR), 30 ms; number of excitations, 1; flip angle, 45°; field of view (FOV), 22 × 22 cm2; and matrix size, 256 × 256 pixels. In the whole-brain DT-MRI examination, sets of axial diffusion-weighted, single-shot echo-planar images were collected with the following parameters: 128 × 128 acquisition matrix, 1.72 × 1.72 × 2 mm3 voxels; 70 axial slices; FOV, 22 × 22 cm2; TE, 60 ms; TR, 7696 ms; flip angle, 90°; slice gap, 0 mm; and b-factor, 600 s/mm–2. Diffusion-weighted images were acquired from 45 different directions using the baseline image without weighting [0, 0, 0]. All axial sections were acquired parallel to the intercommissural (anterior/posterior commissure) line.

### Comparison with previous methods

In order to assess the performance of the SGDM method, we experimented with all 38 subjects comparing the results of the NN, Linear, and SGDM methods in each registration algorithm. First, we investigated regional differences in the MD values using paired Student's *t*-tests. Differences in MD values among the three methods can be used to evaluate the accuracy of the mapping methods indirectly. This can evaluate which method shows the patterns of MD values in the cortical GM similar to those known from previous studies. Diffusion smoothing with a full-width half-maximum of 10 mm was used to blur each map of the MD value, which increased both the signal-to-noise ratio and the statistical power. The cortical surface model contained 81,924 vertices; therefore, correction for multiple comparisons was performed by random field theory at a corrected probability value of 0.05 (Worsley et al., 1996). Second, we projected the b0 tissue classification images to the cortical mid surface using all the three methods and calculated the probability of the CSF class in each vertex to evaluate the accuracy of each mapping method. If mapping is performed inaccurately, the CSF tissue class in the volume space can be projected to the mid surface vertex. The b0 tissue classification was performed using FMRIB's Automated Segmentation Tool (Zhang et al., 2001). Because of the poor contrast between the GM and WM in the b0 volume, we divided it into two classes (CSF, and brain matter = GM + WM). The information regarding tissue classification at the vertices was then transformed to a standard space after projecting it to the mid surface. To evaluate the robustness of SGDM method, we compared the results of SGDM method with ANTs-SyN registration to the result of SGDM method with affine registration. The multiple comparisons were corrected by random field theory at a corrected probability value of 0.05.

## Results

### Regional pattern of MD values

**Figure 4:**
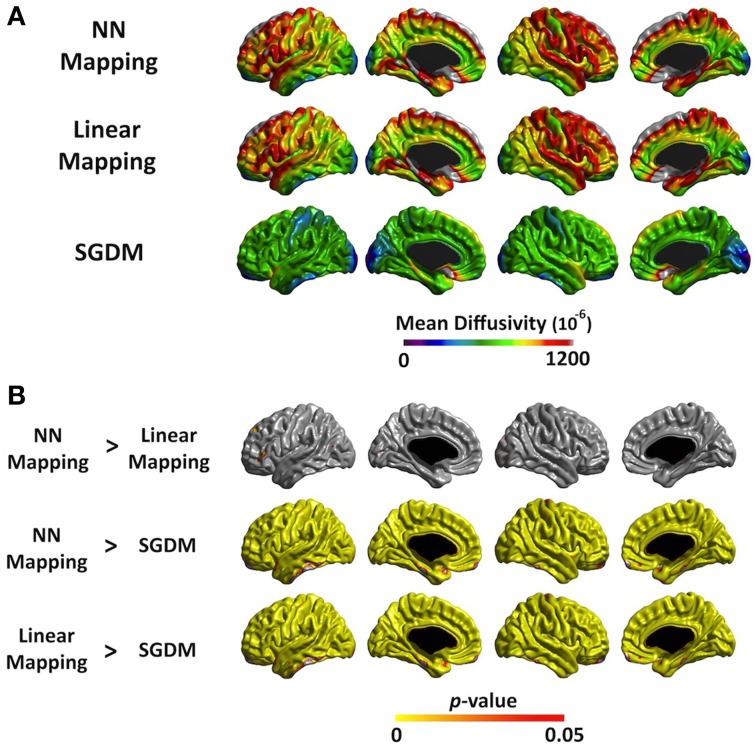
**(A)** The average MD value map for NN, Linear, and SGDM method in affine registration. The color scale on the bottom represents the MD values with a range of 0–1200^−6^. **(B)** Statistical maps of the differences in MD values among the three methods in affine registration. The color scale on the bottom represents the multiple comparison corrected *p* < 0.05.

**Figure 5:**
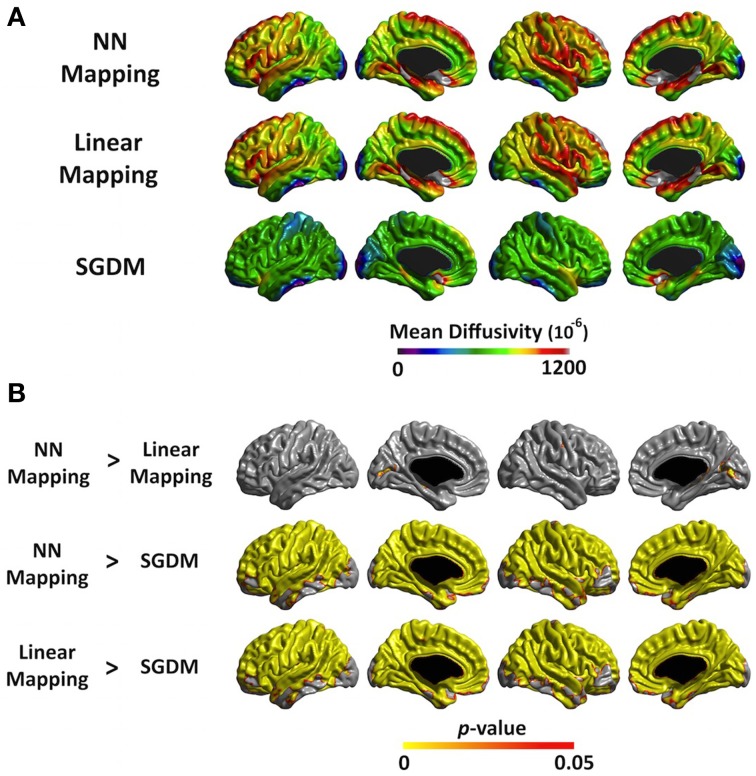
**(A)** The average MD value map for NN, Linear and SGDM method in ANTs-SyN registration. The color scale on the bottom represents the MD values with a range of 0–1200^−6^. **(B)** Statistical maps of the differences in MD values among the three methods in ANTs-SyN registration. The color scale on the bottom represents the multiple comparison corrected *p* < 0.05.

The mean MD values of the entire cortex estimated by Linear and NN methods were significantly greater than that by the SGDM method (*P* < 0.001), but there was no significant difference between the first two methods in both of registration algorithms. Figure 4A showed the regional pattern of MD values for the three approaches in affine registration, where the frontal lobe and, superior temporal lobe and hippocampal gyrus showed the greatest divergence among them. The MD values for the occipital lobe and inferior temporal were small and those for the para-hippocampal, prefrontal regions and medial frontal regions were large for all three methods. As shown in Figure 4B, the MD values of the SGDM method were significantly lower than those of the NN and Linear methods for most of regions and the MD values of the NN and Linear methods were not significantly different in the most of regions. Figure 5A showed the regional pattern of MD values for the three approaches in ANTs-SyN registration, where the frontal lobe and, superior temporal lobe and hippocampal gyrus showed the greatest divergence among them as in affine registration. The MD values for the occipital lobe and inferior temporal were small and those for the para-hippocampal, prefrontal regions and medial frontal regions were large for all three methods. As shown in Figure 5B, the MD values of the SGDM method were significantly lower than those of the NN and Linear methods for most of regions except for the temporal tip, lateral prefrontal region, inferior temporal and occipital regions and the MD values of the NN and Linear methods were not significantly different in the most of regions. As illustrated in **Figure 7**, relative to the MD value of SGDM with ANTs-SyN, those with affine registration decreased in the bilateral inferior temporal lobe and internal capsule and increased in pre-cuneus, lingual, fusiform gyrus, and right inferor parietal region.

### Probability of being classified as CSF

**Figure 6:**
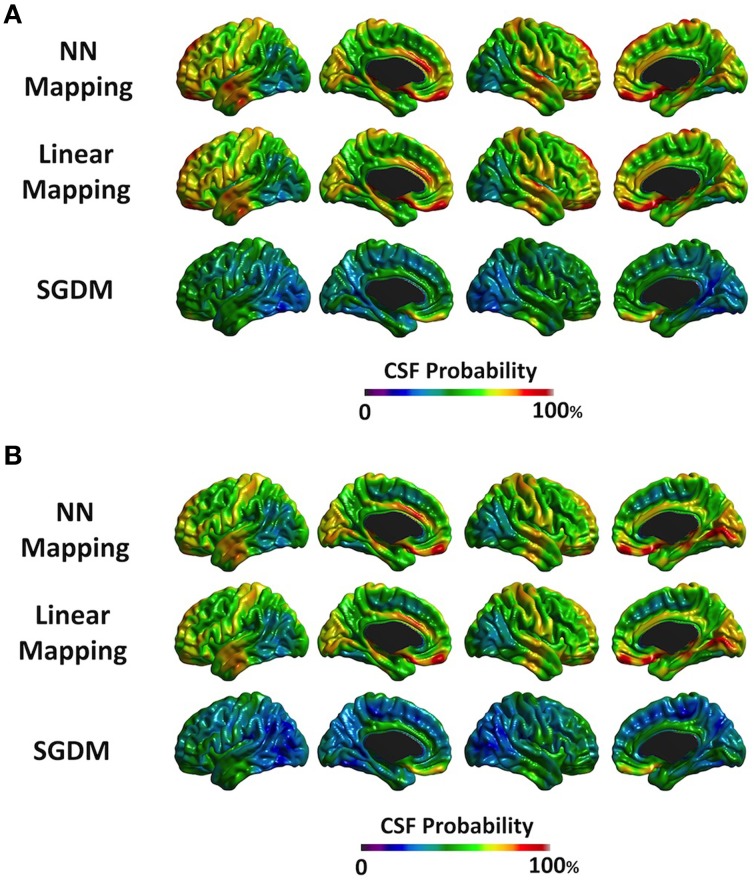
**The probability map of the CSF tissue class**. The color scale on the bottom represents the probability of being classified as CSF. **(A)** The results of ANTs-SyN registration. **(B)** The results of affine registration.

Figure 6 showed the surface mapping of CSF class. Figure 6A is the results of SGDM with ANTs-SyN registration. Figure 6B is the results of SGDM with affine registration. The mean probabilities of CSF class were 59.9% for affine registration and 57.2% for ANTs-SyN registration in NN method. The mean probabilities were 56.1% for affine registration and 46.7% for ANTs-SyN registration in Linear method. The mean probabilities were 39.7% for affine registration and 35.2% for ANTs-SyN registration in SGDM method. The NN and Linear methods showed ~70% of the probability of CSF in the superior part of the brain, prefrontal region, and anterior cingulate region, and over 40% in most of the gyri in both of registration algorithms. On the contrary, the SGDM method showed less than 15% of the probability of CSF in most regions apart from the prefrontal region (80% probability) and the inferior part of the temporal lobe (over 70% probability) in both of registration algorithm.

## Discussion

### Elevated MD values in the NN and linear methods

In this study, we found that the MD values were significantly increased in Linear and NN methods in both of registration algorithm, but there was no significant difference between the first two methods in the most of regions. The mean MD values of the NN and Linear methods were larger than that of SGDM by about 2.0 × 10^−3^ mm^2^/s. Koo et al. (2009) reported that the MD value in the cortical GM was generally ranging between 6.0 ~ 7.5 × 10^−3^ mm^2^/s, and the measured MD value contaminated by CSF was ranging between 8.0 ~ 9.0 × 10^−3^ mm^2^/s (Koo et al., 2009). The MD value measured by SGDM in cortical GM was similar to the previous studies (Latour and Warach, 2002; Liu et al., 2006; Koo et al., 2009), but the MD values measured by the NN and Linear methods were above 10.0 × 10^−3^ mm^2^/s.

### Regional pattern differences of mapping methods

**Figure 7:**
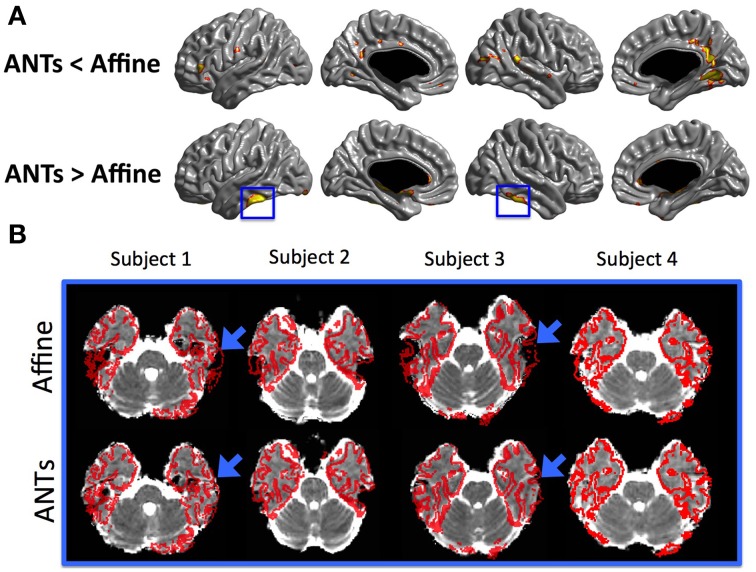
**(A)** Statistical maps of the differences in MD values between the SGDM with ANTs-SyN and the SGDM with affine registration. The color scale on the bottom represents the multiple comparison corrected *p* < 0.05. **(B)** The cortical surface superimposed in the MD volume. The MD volume was aligned to T1-wighted volume space using affine registration and ANTs-SyN registration. We choose the 4 subjects. The blue rectangles in **(A)** correspond to the location of blue arrow in **(B)**.

The regional pattern of MD value in cortical GM is also consistent with the previous studies. Koo et al. (2009) also revealed that the MD values for superior parietal region and occipital region were smaller and the para-hippocampal and medial frontal regions were larger than the other region (Koo et al., 2009). The pattern of MD value in SGDM was consistent with the previous study, but not in NN and Linear methods in both of registration algorithms. These results indicated that Linear and NN methods were more influenced by the MD value in CSF region. Linear and NN methods projected the MD value in CSF region, which results in the overestimation of the true MD value. As shown in Figure 6, the superior parts of the brain, that is, the post-central, anterior cingulate, and temporal lobe, showed a high probability of CSF for the NN and Linear methods in both of registration algorithm. The probability of being classified as CSF by the SGDM approach was lower than by the NN and Linear methods in most regions in both of registration algorithm. The high probability of being classified as CSF in the NN and Linear methods resulted in higher MD values for these methods than in the SGDM approach. The post-central, anterior cingulate, and temporal lobe is known to suffer from CSF contamination effect and geometric distortion (Jezzard and Balaban, 1995). This makes it difficult to achieve accurate coregistration between the b0 and the T1-weighted volumes (Jezzard and Balaban, 1995). The results suggested that such inaccuracy could lead to a mapping error that assigns the MD value in the CSF region to the midsurface (GM region). Overall consistency between the MD value in SGDM and the MD value measured by fluid-attenuated inversion recovery (FLAIR) DTI in the previous study indicated that the proposed SGDM technique would be adequate for handling the mapping error caused by mis-alignment between the b0 and T1-weighted volume. The high probability of CSF in the prefrontal regions still existed even when the SGDM approach was used. The prefrontal region is known to be influenced by the geometric distortion more severely than the other regions because of their close proximity to the cranial sinus cavities (Cusack et al., 2003). Because the prefrontal region suffers from non-linear geometric distortion (Jezzard and Balaban, 1995), even the SGDM cannot work accurately in this area. The MD values in the occipital poles were the lowest across the cortex for all the three methods (Figure 4). The low MD values in this region could be caused by surface generation errors resulting from the low contrast of GM and WM in the T1-weighted volume, or caused by the particular cytoarchitecture of the cortex and its high level of myelination (Steen et al., 2000). Because the SGDM defines the profile direction from the voxel overlaid with the vertex of the pial surface toward the line followed by the cortical column, the performance of the SGDM depends on the accuracy of the estimated cortical surface. If the surface is not well established, the performance of the SGDM will be unsatisfactory. If we could mitigate the problem of low tissue contrast in the occipital region by using a different tissue segmentation algorithm or improved T1-weighted acquisition, surface generation might be improved and the performance of SGDM could be improved for this region. In this study, we proposed the SGDM method to project the MD value to the cortical surface without mapping errors caused by the imperfect coregistration. The aim of this study is to correct the mapping errors and maintain consistency regardless of which registration method we used. As depicted in Figure 7A, our procedure works well with various registration methods. The MD value for two registration methods was not significantly different in the most of regions despite the registration algorithm was not identical. The result indicates the SGDM method would effectively correct mapping error and maintain the performance. Nevertheless, relative to the MD value of SGDM with ANTs-SyN, those with affine registration decreased in the bilateral inferior temporal lobe and internal capsule and increased in pre-cuneus, lingual, fusiform gyrus and right inferor parietal region as shown in Figure 7A (blue rectangle). The significant difference might result from coregistration performance in these regions. As illustrated in Figure 7B, contrary to the ANTS-SyN affine registration, the cortical surface (red dots) is not well overlaid with MD volume (blue arrow) in affine registration. In these circumstances, the zero value was projected to the vertex of mid-surface as shown in Figure 7B (blue arrow). However, the misalignment in the ANTs-SyN algorithms is less severe than those in affine registration. The MD value larger than zero was projected. Therefore, these significant differences would be the result of the large misalignment between two objects caused by geometric distortion. If the geometric distortion is severe, then these areas will be misregistered. When the brain is has large geometric distortion, affine registration would not be sufficient, whereas the high-order warping such as ANTs-SyN could be an alternative. The essential element to SGDM's performance is that it is based on an assumption that the mapping error can be corrected by shifting linearly along the MD profile. As shown in Figure 7B, the cortical surface is not well overaid with MD volume in affine registration. The MD profile in these regions would not be drawn successfully in affine registration. These mean that the affine registration is insufficient to satisfy the assumption. Therefore, we recommend that the researcher performs coregistration using a fine registration algorithm, then use SGDM method. Even if the geometric distortion is not severe, the use of high-order warping such as ANTs-SyN could improve the performance of SGDM.

### Advantages

The SGDM algorithm has two advantages over previous approaches. Firstly, it does not need any additional field map acquisition for minimizing the geometric distortion that leads to faulty assignment of MD values. The SGDM method also makes it possible to analyze MD values in the cortical GM compared with other measurements (such as cortical thickness or sulcal depth) to examine the relationship between micro- and macrostructural changes. Furthermore, the SGDM approach allows the use of the partial volume fraction map generated from the T1-weighted volume to correct for contamination by CSF. Because the T1-weighted volume has better tissue contrast than the b0 volume, we were able to estimate the PVE of CSF more accurately.

## Conclusion

We propose a novel method called SGDM to correct mapping errors using the cortical surface model generated from the T1-weighted volume. The faulty assignment of the MD values caused by affine or rigid-body coregistration was minimized successfully using this approach on conventional DTI and T1-weighted sequences. Our results indicate that the SGDM approach is better than the NN and Linear methods in the absence of additional information about geometric distortions, such as a field map. Our results suggest that the fine registration algorithm such as ANTs-SyN could improve the performance of SGDM. Because of the poor contrast in the b0 volume, we divided it into two classes (CSF and brain matter) for evaluation. If we could divide the b0 volume into three classes (i.e., CSF, GM, and WM), better evaluation should be possible.

### Conflict of interest statement

The authors declare that the research was conducted in the absence of any commercial or financial relationships that could be construed as a potential conflict of interest.

## References

[B1] Avants B. B. Epstein C. L. Grossman M. Gee J. C. (2008). Symmetric diffeomorphic image registration with cross-correlation: evaluating automated labeling of elderly and neurodegenerative brain. Med. Image Anal. 12, 26–41. 10.1016/j.media.2007.06.004 17659998 PMC2276735

[B2] Basser P. J. (1995). Inferring microstructural features and the physiological state of tissues from diffusion-weighted images. NMR Biomed. 8, 333–344. 10.1002/nbm.1940080707 8739270

[B3] Beaulieu C. (2002). The basis of anisotropic water diffusion in the nervous system - a technical review. NMR Biomed. 15, 435–455. 10.1002/nbm.782 12489094

[B4] Brett M. Leff A. P. Rorden C. Ashburner J. (2001). Spatial normalization of brain images with focal lesions using cost function masking. Neuroimage 14, 486–500. 10.1006/nimg.2001.0845 11467921

[B5] Càmara E. Bodammer N. Rodríguez-Fornells A. Tempelmann C. (2007). Age-related water diffusion changes in human brain: a voxel-based approach. Neuroimage 34, 1588–1599. 10.1016/j.neuroimage.2006.09.045 17188516

[B6] Chung M. K. Worsley K. J. Robbins S. Paus T. Taylor J. (2003). Deformation-based surface morphometry applied to gray matter deformation. Neuroimage 18, 198–213. 10.1016/S1053-8119(02)00017-4 12595176

[B7] Collins D. L. Neelin P. Peters T. M. Evans A. C. (1994). Automatic 3D intersubject registration of MR volumetric data in standardized Talairach space. J. Comput. Assist. Tomogr. 18, 192–205. 10.1097/00004728-199403000-00005 8126267

[B8] Crum W. R. Griffin L. D. Hill D. L. G. Hawkes D. J. (2003). Zen and the art of medical image registration: correspondence, homology, and quality. Neuroimage 20, 1425–1437. 10.1016/j.neuroimage.2003.07.014 14642457

[B9] Cusack R. Brett M. Osswald K. (2003). An evaluation of the use of magnetic field maps to undistort echo-planar images. Neuroimage 18, 127–142. 10.1006/nimg.2002.1281 12507450

[B10] Fellgiebel A. Wille P. Müller M. J. Winterer G. Scheurich A. Vucurevic G.. (2004). Ultrastructural hippocampal and white matter alterations in mild cognitive impairment: a diffusion tensor imaging study. Dement. Geriatr. Cogn. Disord. 18, 101–108. 10.1159/000077817 15087585

[B11] Gartus A. Geissler A. Foki T. Tahamtan A. R. Pahs G. Barth M.. (2007). Comparison of fMRI coregistration results between human experts and software solutions in patients and healthy subjects. Eur. Radiol. 17, 1634–1643. 10.1007/s00330-006-0459-z 17036153

[B12] Hutton B. F. Braun M. (2003). Software for image registration: algorithms, accuracy, efficacy. Semin. Nucl. Med. 33, 180–192. 10.1053/snuc.2003.127309 12931320

[B13] Hutton C. Bork A. Josephs O. Deichmann R. Ashburner J. Turner R. (2002). Image distortion correction in fMRI: a quantitative evaluation. Neuroimage 16, 217–240. 10.1006/nimg.2001.1054 11969330

[B14] Jenkinson M. Bannister P. Brady M. Smith S. (2002). Improved optimization for the robust and accurate linear registration and motion correction of brain images. Neuroimage 17, 825–841. 10.1006/nimg.2002.1132 12377157

[B15] Jezzard P. Balaban R. S. (1995). Correction for geometric distortion in echo planar images from B0 field variations. Magn. Reson. Med. 34, 65–73. 10.1002/mrm.1910340111 7674900

[B16] Kim J. S. Singh V. Lee J. K. Lerch J. Ad-Dab'bagh Y. MacDonald D.. (2005). Automated 3-D extraction and evaluation of the inner and outer cortical surfaces using a laplacian map and partial volume effect classification. Neuroimage 27, 210–221. 10.1016/j.neuroimage.2005.03.036 15896981

[B17] Klein A. Andersson J. Ardekani B. A. Ashburner J. (2009). Evaluation of 14 nonlinear deformation algorithms applied to human brain MRI registration. Neuroimage 46, 786–802. 10.1016/j.neuroimage.2008.12.037 19195496 PMC2747506

[B18] Koo B.-B. Hua N. Choi C.-H. Ronen I. Lee J. M. Kim D.-S. (2009). A framework to analyze partial volume effect on gray matter mean diffusivity measurements. Neuroimage 44, 136–144. 10.1016/j.neuroimage.2008.07.064 18775785

[B19] Kubicki M. Westin C.-F. Maier S. E. Mamata H. Frumin M. Ersner-Hershfield H.. (2002). Diffusion tensor imaging and its application to neuropsychiatric disorders. Harv. Rev. Psychiatry 10, 324–336. 10.1080/10673220216231 12485979 PMC2853779

[B20] Latour L. L. Warach S. (2002). Cerebral spinal fluid contamination of the measurement of the apparent diffusion coefficient of water in acute stroke. Magn. Reson. Med. 48, 478–486. 10.1002/mrm.10238 12210912

[B21] Lerch J. P. Evans A. C. (2005). Cortical thickness analysis examined through power analysis and a population simulation. Neuroimage 24, 163–173. 10.1016/j.neuroimage.2004.07.045 15588607

[B22] Liu T. Young G. Huang L. Chen N.-K. Wong S. T. C. (2006). 76-space analysis of gray matter diffusivity: methods and applications. Neuroimage 31, 51–65. 10.1016/j.neuroimage.2005.11.041 16434215

[B23] MacDonald D. Kabani N. Avis D. Evans A. C. (2000). Automated 3-D extraction of inner and outer surfaces of cerebral cortex from MRI. Neuroimage 12, 340–356. 10.1006/nimg.1999.0534 10944416

[B24] Moseley M. E. Wendland M. F. Kucharczyk J. (1991). Magnetic resonance imaging of diffusion and perfusion. Top. Magn. Reson. Imaging 3, 50–67. 2054198

[B25] Naggara O. Oppenheim C. Rieu D. Raoux N. Rodrigo S. Dalla Barba G.. (2006). Diffusion tensor imaging in early Alzheimer's disease. Psychiatry Res. 146, 243–249. 10.1016/j.pscychresns.2006.01.005 16520023

[B26] Ray K. M. Wang H. Chu Y. Chen Y.-F. Bert A. Hasso A. N.. (2006). Mild cognitive impairment: apparent diffusion coefficient in regional gray matter and white matter structures. Radiology 241, 197–205. 10.1148/radiol.2411051051 16990677

[B27] Robbins S. M. (2003). Anatomical Standardization of the Human Brain in Euclidean 3-space and on the Cortical 2-Manifold. Ph.D. thesis, School of Computer Science, McGill University, Montreal, QC.

[B28] Rose S. E. Janke A. L. Chalk J. B. (2008). Gray and white matter changes in alzheimer's disease: a diffusion tensor imaging study. J. Magn. Reson. Imaging 27, 20–26. 10.1002/jmri.21231 18050329

[B29] Saad Z. S. Glen D. R. Chen G. Beauchamp M. S. Desai R. Cox R. W. (2009). A new method for improving functional-to-structural MRI alignment using local pearson correlation. Neuroimage 44, 839–848. 10.1016/j.neuroimage.2008.09.037 18976717 PMC2649831

[B30] Serra L. Cercignani M. Lenzi D. Perri R. Fadda L. Caltagirone C.. (2010). Grey and white matter changes at different stages of Alzheimer's disease. J. Alzheimers Dis. 19, 147–159. 10.3233/JAD-2010-1223 20061634

[B31] Sled J. G. Zijdenbos A. P. Evans A. C. (1998). A nonparametric method for automatic correction of intensity nonuniformity in MRI data. IEEE Trans. Med. Imaging 17, 87–97. 10.1109/42.668698 9617910

[B32] Smith S. M. (2002). Fast robust automated brain extraction. Hum. Brain Mapp. 17, 143–155. 10.1002/hbm.10062 12391568 PMC6871816

[B33] Smith S. M. Jenkinson M. Woolrich M. W. Beckmann C. F. Behrens T. E. J. Johansen-Berg H. (2004). Advances in functional and structural MR image analysis and implementation as FSL. Neuroimage 1, S208–S219. 10.1016/j.neuroimage.2004.07.051 15501092

[B34] Steen R. G. Reddick W. E. Ogg R. J. (2000). More than meets the eye: significant regional heterogeneity in human cortical T1. Magn. Reson. Imaging 18, 361–368. 10.1016/S0730-725X(00)00123-5 10788712

[B35] Sundgren P. C. Dong Q. Gómez-Hassan D. Mukherji S. K. Maly P. Welsh R. (2004). Diffusion tensor imaging of the brain: review of clinical applications. Neuroradiology 46, 339–350. 10.1007/s00234-003-1114-x 15103435

[B36] Tohka J. Zijdenbos A. Evans A. (2004). Fast and robust parameter estimation for statistical partial volume models in brain MRI. Neuroimage 23, 84–97. 10.1016/j.neuroimage.2004.05.007 15325355

[B37] Turken A. U. Herron T. J. Kang X. O'Connor L. E. Sorenson D. J. Baldo J. V.. (2009). Multimodal surface-based morphometry reveals diffuse cortical atrophy in traumatic brain injury. BMC Med. Imaging 9:20. 10.1186/1471-2342-9-20 20043859 PMC2811103

[B38] Villain N. Landeau B. Groussard M. Mevel K. Fouquet M. Dayan J.. (2010). A simple way to improve anatomical mapping of functional brain imaging. J. Neuroimaging 20, 324–333. 10.1111/j.1552-6569.2010.00470.x 20331499 PMC3021536

[B39] Vrenken H. Pouwels P. J. W. Geurts J. J. G. Knol D. L. Polman C. H. Barkhof F.. (2006). Altered diffusion tensor in multiple sclerosis normal-appearing brain tissue: cortical diffusion changes seem related to clinical deterioration. J. Magn. Reson. Imaging 23, 628–636. 10.1002/jmri.20564 16565955

[B40] Worsley K. J. Marrett S. Neelin P. Vandal A. C. Friston K. J. Evans A. C. (1996). A unified statistical approach for determining significant signals in images of cerebral activation. Hum. Brain Mapp. 4, 58–73. 10.1002/(SICI)1097-0193(1996)4:1<58::AID-HBM4>3.0.CO;2-O 20408186

[B41] Zhang Y. Brady M. Smith S. (2001). Segmentation of brain MR images through a hidden markov random field model and the expectation-maximization algorithm. IEEE Trans. Med. Imaging 20, 45–57. 10.1109/42.906424 11293691

[B42] Zhou F. Zee C.-S. Gong H. Shiroishi M. Li J. (2010). Differential changes in deep and cortical gray matters of patients with multiple sclerosis: a quantitative magnetic resonance imaging study. J. Comput. Assist. Tomogr. 34, 431–436. 10.1097/RCT.0b013e3181cbf73c 20498549

[B43] Zijdenbos A. P. Forghani R. Evans A. C. (2002). Automatic “pipeline” analysis of 3-D MRI data for clinical trials: application to multiple sclerosis. IEEE Trans. Med. Imaging 21, 1280–1291. 10.1109/TMI.2002.806283 12585710

[B44] Zilles K. Armstrong E. Schleicher A. Kretschmann H. J. (1988). The human pattern of gyrification in the cerebral cortex. Anat. Embryol. 179, 173–179. 10.1007/BF00304699 3232854

